# Nanocomposite Bone Scaffolds Based on Magnesium Alloy: A Detailed Investigation of Their In‐Vitro Biodegradation Performance

**DOI:** 10.1155/ijbm/9617232

**Published:** 2026-02-08

**Authors:** Adithya Garimella, Firoz Alam Faroque, Subrata Bandhu Ghosh, Sanchita Bandyopadhyay-Ghosh

**Affiliations:** ^1^ Department of Mechanical and Industrial Engineering, Manipal Institute of Technology Bengaluru, Manipal Academy of Higher Education, Manipal, 576104, Karnataka, India, manipal.edu; ^2^ Department of Civil Engineering, Manipal Institute of Technology Bengaluru, Manipal Academy of Higher Education, Manipal, 576104, Karnataka, India, manipal.edu; ^3^ Department of Mechanical Engineering, Engineered Biomedical Materials Research and Innovation Centre (EnBioMatRIC), Manipal University Jaipur, Jaipur, 303007, Rajasthan, India, manipal.edu

**Keywords:** biodegradable metallic-based bone scaffold, biodegradation, nanocomposite scaffold, porous magnesium-based alloy

## Abstract

Polymeric biomaterials and their composites have been extensively explored for orthopaedic applications; however, their inadequate mechanical performance significantly restricts their use in load‐bearing environments. Metallic biomaterials, by contrast, offer superior mechanical strength and structural stability. Among them, magnesium (Mg) has emerged as a particularly attractive candidate for temporary orthopaedic implants owing to its elastic modulus and density being close to those of natural bone, thereby minimising stress shielding. In addition, Mg inherently fulfils two critical requirements for orthopaedic implants—biocompatibility and biodegradability. In this study, a bioactive magnesium‐alloy‐based nanocomposite scaffold was engineered to overcome the limitations of conventional biomaterials while closely replicating the porous microarchitecture of human bone. A novel bioactive glass–ceramic, nano‐fluorcanasite (n‐FC), was incorporated into the Mg‐alloy matrix to enhance osteogenic activity and accelerate bone tissue regeneration. The introduction of an interconnected porous structure was designed to promote efficient nutrient diffusion, facilitate metabolic waste removal and reduce implant density. Furthermore, the controlled addition of selected alloying elements in specific weight fractions effectively moderated the degradation kinetics of the Mg‐based scaffold. The nanocomposite scaffolds were fabricated using a powder metallurgy route followed by sintering. Tailored porosity was achieved through the controlled incorporation of carbamide particles as a space‐holding agent. The in vitro degradation behaviour of the scaffolds was systematically evaluated using a weight‐change method after immersion in phosphate‐buffered saline (PBS) for predetermined durations. The results demonstrate that, compared with unalloyed magnesium, the degradation rate of the nanocomposite scaffolds can be precisely and consistently regulated, highlighting their potential as mechanically competent, bioactive and biodegradable candidates for orthopaedic implant applications.

## 1. Introduction

Bone diseases, traumas and fractures have emerged as significant concerns due to the increasing average age of the global population. These bone damages, fractures and numerous skeletal‐related problems may be addressed by the use of the bone transplant–graft approach, which is a standard strategy that is often performed [1]. Many investigators were motivated to develop scaffolds composed of next‐generation materials in the field of bone tissue engineering (BTE) due to the significant constraints of current bone transplantation methods [2]. The increase in the incidence of bone fractures and injuries affecting both people and animals in recent decades has led to a significant growth in the demand for biomaterial implants [3]. Annually, over 10 million bone scaffolds are used in bone‐repair procedures globally. The primary purpose of these implants is to expedite up the process of new bone regeneration and bone repair, which is the process of replacing broken and diseased bones that have been caused by infection, injury or illness [4]. BTE necessitates the construction of a scaffold with a three‐dimensional structure that has characteristics such as porosity, biocompatibility and biodegradability [5]. Bone implants that feature a porous design has reduced self‐weight and the presence of extensively interconnected pores, which significantly enhance the exchange of oxygen and nutrients, both essential for blood circulation during the regeneration of new bone cells [6, 7].

A wide range of scaffolding biomaterials, including ceramics, metals and polymers, have become prevalent in biomedical applications. Ceramic biomaterials are biocompatible; but, owing to their barely enough mechanical characteristics, they are just appropriate for restricted load bearing applications [8, 9]. There are barriers to the uses of polymeric scaffolds resulting from their paucity of biocompatible feature and biodegradable nature. It is possible to avoid these limitations by using scaffolds made of metallic materials that have needed mechanical specs and include a biocompatibility feature, which positions them as a formidable candidate for load bearing applications [10, 11]. Nonetheless, the use of these metallic‐based scaffolds is limited by their susceptibility to corrosion and the release of detrimental metallic ions [12, 13]. The applications of regularly used metallic biomaterials in bone implants are limited due to the constraints that are caused by the differences in numerous mechanical characteristics between the natural hard tissue and materials, besides the stress‐shielding effect [14, 15].

Researchers have focused on exploring metallic biodegradable materials because of the limitations of current scaffolds. Magnesium (Mg) is classified as one of these materials. Considering its mechanical characteristics, magnesium has been proven to be comparable to those of actual human bone [7]. On the other hand, one of the most significant concerns regarding magnesium is the higher degradation that occurs in saline as a consequence of the inclusion of free ions of chlorine [16, 17]. The addition of particular alloying elements to the magnesium metal matrix may address the constraints of scaffolds made from magnesium material. In this context, metals such as calcium (Ca), zinc (Zn) and strontium (Sr), for instance, have exhibited as a great substitute for traditional alloying elements. Ca, which is a significant component of bone, has the ability to stimulate bone formation [18, 19]. Inclusion of around 2 wt% of Ca into Mg metal matrix is anticipated to result in the formation of a layer of calcium phosphate over the surface, which could possibly improve the resistance towards degradation [20–22]. However, introduction of Zn into the Mg–Ca metal matrix could potentially enhance the corrosion resistance and mechanical properties, as Zn is a grain refiner and could also encourage in solid solution strengthening. Zn when incorporated at 3 wt% into the Mg–Ca metal matrix possibly will decrease the rates of degradation as due to the existence of α‐Mg matrix and γ‐Mg–Zn phase and could also enhance their *in vitro* and *in vivo* performance of the Mg–Ca–Zn alloy system [23, 24]. On the other hand, when lower amounts of Sr (0.5 wt%) is added into the Mg–Ca–Zn metal matrix, it is expected to activate the electroconductivity into the alloy system [25]. Hence, this work adopts a synergistic multi‐alloying strategy involving Mg–Ca–Zn–Sr to develop biodegradable scaffolds with controlled degradation behaviour. By selectively incorporating exclusively osteogenic alloying elements into the magnesium matrix, the proposed biomimetic alloy design enables enhanced mechanical performance, bioactivity‐driven surface mineralisation and electroconductive functionality. The resulting Mg–Ca–Zn–Sr scaffold system represents a novel approach towards multifunctional biodegradable implants for orthopaedic applications. Accordingly, the primary objective of this research is to develop a controlled, biodegradable and biocompatible magnesium‐based scaffold through the strategic incorporation of selected alloying elements.

## 2. Materials and Methods

### 2.1. Materials

The specifications of the preliminary materials, which include the purity, the average particle size and the manufacturer, are mentioned in Table 1.

**TABLE 1:** Particulars about the ingredients that were used in the fabrication of the sample.

S. no.	Material	Mean size of the particle	Purity (%)	Manufacturer
1	Magnesium	40–60 μm	≥ 99.5	Alfa Aesar, USA
2	Calcium	45–60 μm	≥ 98.5	HiMedia, India
3	Zinc	30–50 μm	≥ 99.5	HiMedia, India
4	Strontium	30–40 μm	≥ 99.0	HiMedia, India
5	Fluorcanasite	80–90 nm	≥ 99.0	*In-house*
6	Urea	150–200 μm	≥ 99.0	HiMedia, India

As summarised, in Table 1, all the preliminary materials were procured from various sources, while, fluorcanasite (K_3_Na_3_Ca_5_Si_12_O_30_F_4_H_2_O) was synthesised in‐house (at ‘Engineered Biomedical Materials Research and Innovation Centre’ (EnBioMatRIC), Manipal University Jaipur, India). The synthesis procedure was executed in line with the methodology previously established by our research team [26]. In outline, the method comprises melting glass compositions with specific proportions of oxides at a temperature of 1450°C in a raising hearth furnace or bottom loading furnace (AP‐BLF‐1400‐X Ants lab). The glass frits were subjected to ball milling in a planetary ball mill (*E*_max_, Retsch) for a duration of 50 h at a speed of 900 rpm. This process resulted in the formation of glass nano‐particulates. Subsequently, a two‐stage controlled heat treatment was conducted on the fine glass particles to transform them into nano‐fluorcanasite (n‐FC) glass–ceramic particles.

### 2.2. Development of the Mg‐Alloy‐Based Bio‐Nanocomposite Bone Scaffold Sample

‘Press and sinter’ process, which is also called as powder metallurgy technique, was employed to manufacture the scaffold specimens, as per the compositions listed in Table 2.

**TABLE 2:** Specifics of various developed bio‐nanocomposite samples.

Code for the developed porous sample		Mg (weight %)	Ca (weight %)	Zn (weight %)	Sr (weight %)	n‐FC (weight %)	Intended porosity (%)
Control samples	C30	100	0	0	0	0	30
	C45	100	0	0	0	0	45
	C60	100	0	0	0	0	60

Mg‐alloy samples	X30	93.5	1	2.5	0.5	2.5	30
	X45	93.5	1	2.5	0.5	2.5	45
	X60	93.5	1	2.5	0.5	2.5	60
	Y30	91.5	3	2.5	0.5	2.5	30
	Y45	91.5	3	2.5	0.5	2.5	45
	Y60	91.5	3	2.5	0.5	2.5	60

**FIGURE 1:**
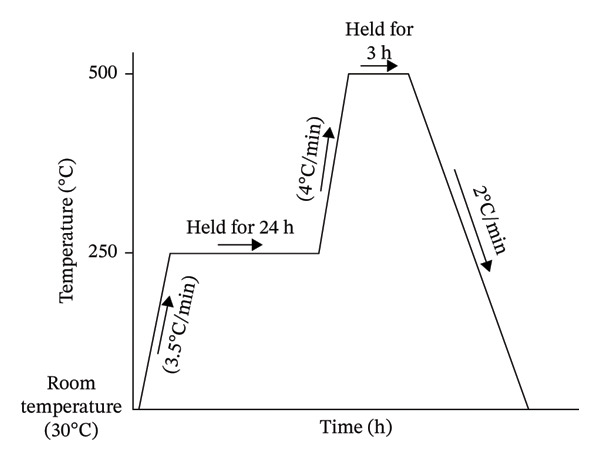
Profile of the heat treatment that was employed during the two‐step sintering procedure.

**FIGURE 2:**
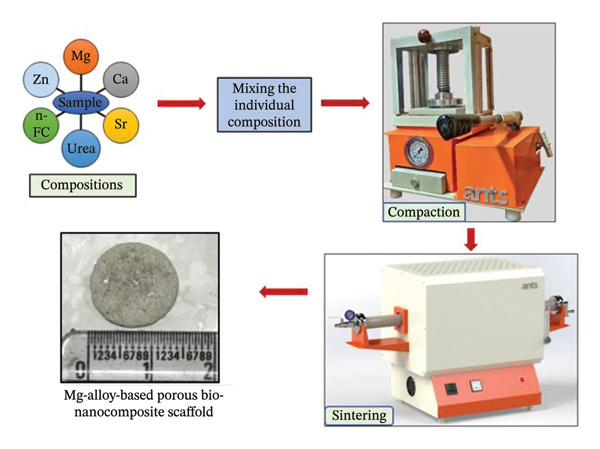
Step‐by‐step process employed to fabricate the magnesium‐alloy‐based bio‐nanocomposite porous scaffold specimen.

Agate crusher was used for disintegrating the gritty metallic‐calcium fragments into a finely divided powder, which was then sieved using a 50‐micron sieve in order to make certain that the particle size was consistent throughout. A tumbler‐mixer arrangement was employed, which combines these fine particulates in accordance with the various compositions that are exhibited in Table 2. To induce the aimed porosity of 30%, 45% and 60% individually in the scaffold specimens, an equivalent amount (in weight) was incorporated into the respective compositions. The fabricated samples were designed to exhibit porosities of 30%, 45% and 60%, which were achieved by incorporating corresponding volumetric fractions of carbamide as the pore‐forming agent. According to Table 2, distinct and unique composition of scaffold specimens was obtained by weighing the precursor materials individually. These discrete compositional mixtures were thoroughly blended and were uniaxially compressed (using a pellet die made of tungsten carbide), thereby achieving a thin, three‐dimensional cylindrical structure of diameter 12 × 4 mm height by employing a pressure of 150 MPa using a hydraulic operated press (HPAT20 Ants lab). An inert atmosphere was maintained in the tubular furnace (Ants Prosys 1200°C) by a continuous flow of argon gas (at a rate of ∼ 2 cm^3^/min, such that roughly one argon bubble per second underwater), which resulted in a controlled environment inside the furnace that was employed to perform the sintering operation for the obtained compacted‐compressed scaffold specimens. As can be observed in Figure 1, the scaffold specimens were obtained after a two‐step sintering procedure. The samples were subjected to a temperature of 250°C at a rate of 3.5°C per minute during the initial stage of the procedure. The temperature was then kept constant for a period of 24 h in order to successfully achieve the total evacuation of carbamide particles from the specimen. The specimens were then subjected to an additional heating, during which they rose to a temperature of 500°C at a rate of 4°C/min. The samples were subsequently kept at this temperature for a period of 3 h in order to establish a strong metallic bond between the metal atoms and, therefore, facilitate and encourage the improved densities of the bio‐nanocomposite scaffolds. By using a cooling rate of 2°C/min, the temperature was brought down to the ambient temperature, and thus obtaining a three‐dimensional open porous cylindrical structure. Figure 2 illustrates the step‐by‐step process employed to fabricate the magnesium‐alloy‐based bio‐nanocomposite porous scaffold specimen.

### 2.3. Investigation of In Vitro Degradation Behaviour

#### 2.3.1. Evaluation of Degradation Rate and pH

ASTM‐G31‐72 standards [27] were strictly followed to investigate the *in vitro* biodegradation behaviour of the Mg‐alloy‐based porous bio‐nanocomposite scaffold samples, which were immersed into the phosphate‐buffered saline (PBS) solution for a stipulated time lines. As per these standards, a decrement in the weights of the respective immersed samples in the PBS solution, with a starting pH of 7.4, was observed in this investigation. Post the compaction of the green compacts, sintering operation was performed to obtain porous scaffold samples, as discussed in the earlier section. These obtained Mg‐alloy‐based porous bio‐nanocomposite scaffold samples were uniformly ground up to 2500 grit, to avoid any irregular surfaces, that may influence its biodegradation, post immersion. Sterilised glass vials were employed to submerge the obtained bio‐nanocomposite specimens individually into PBS solution and were keep at 37°C throughout the study. All the scaffold specimens of the three porosities (30%, 45% and 60%) were immersed into the physiological solution for 24, 48, 72, 168 and 360 h, respectively, thereby observing their decrement in the weights against the specified time, so as to calculate the rate of degradation of each sample.

Post the rated time period, these corroded samples were repossessed from the immersion liquid and were cleaned using deionised water (DI). In accordance with ASTM‐G31‐72 standards [27], chromate acid was synthesised, which was used to clean these corroded biocomposite samples. In brief, the chromate acid was prepared by introducing 10 g per litre of silver nitrate with 200 g per litre of chromium trioxide, as per ASTM‐G31‐72 standards. Post cleaning with chromate acid, these degraded porous samples were then gently rinsed with DI water to ensure of removing any unwanted degraded by‐products and residual chromate ions on the surface of the corroded scaffold specimens. The weight of the scaffold samples, both before and after they were immersed in PBS solution, was noted, and this difference of the weights would reveal us the decrement of the mass of the scaffold samples during immersion in the physiological solution; thereby, the degradation rate was computed by the following formula [7]:

(1) degradation rate DR=ΔWCSA×T ,

where Δ*W* (grams) is the weight of the developed composite sample pre‐immersion–weight of the developed composite sample post‐immersion; CSA refers to the surface area of the immersed sample, which was revealed for the physiological liquid and *T* represents the immersion time.

In the surrounding region of the implant, pH is very crucial for the successful bone growth; hence, the pH study is of great importance during the study of *in vitro* degradation behaviour. The pH of the physiological solution was noted by employing an ‘LMPH‐10 Benchtop pH Meter’. During the degradation study, post retrieving the samples from the sample immersion fluid, the pH of the liquid was measured and tabulated. All experimental investigations were carried out in triplicate for all combinations of sample porosities and compositions.

#### 2.3.2. Evaluation of Emission of the Hydrogen Gas

Formation of the hydrogen gas from the immersed Mg‐alloy‐based bio‐nanocomposite scaffold samples is an indication of its rate of biodegradation. During the investigation of the biodegradation examination, the produced sample was located under the inverted funnel with a burette filled with PBS was place over the funnel, thereby, the hydrogen gas emitted from the immersed scaffold sample, can trapped inside the burette. The amounts of hydrogen gas captivated inside the burette can be observed and noted at regular intervals, which were kept in accordance with the time intervals for the degradation study.

## 3. Results and Discussion

### 3.1. Scanning Electron Microscopy of the Starting Materials and Developed Scaffold Samples

**FIGURE 3:**
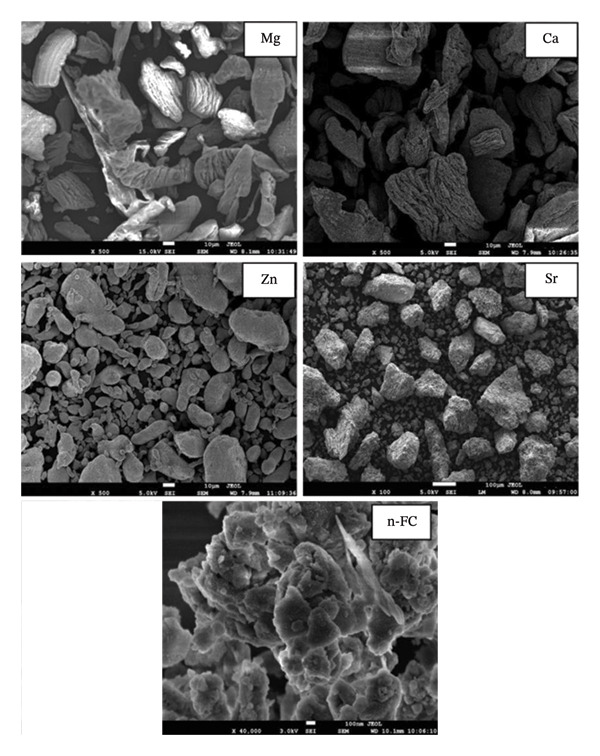
Scanning electron microscope images of the precursor materials that are used to fabricate the scaffold samples.

Figure 3 depicts the images of the scanning electron micrographs of the precursor materials that were employed to develop the Mg‐alloy‐based bio‐nanocomposite scaffold samples. A uniform distribution of Mg, Ca, Zn, Sr and n‐FC particles can be observed from these images. The particle size of these materials was calculated and are tabulated in Table 3. From Table 3, it can be noted that the average particle size of these starting materials is in the same range as claimed in Table 1.

**TABLE 3:** Calculated average particle size of the starting materials obtained from SEM images.

S. no.	Material	Calculated average particle size
1	Magnesium (Mg)	30–60 *µ*m
2	Calcium (Ca)	25–70 *µ*m
3	Zinc (Zn)	30–40 *µ*m
4	Strontium (Sr)	20–50 *µ*m
5	nano‐fluorcanasite (n‐FC)	70–90 nm

**FIGURE 4:**
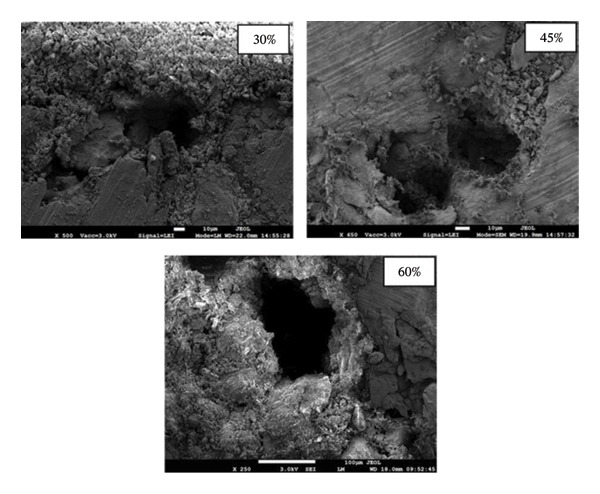
Scanning electron microscope images of the fabricated porous scaffold samples of 30% porosity, 45% porosity and 60% porosity.

Figure 4 depicts the scanning electron microscope images of the developed Mg‐alloy‐based porous bio‐nanocomposite scaffold samples. As observed in Figure 4, increasing porosity led to the development of a highly interconnected pore network within the scaffold samples. Microstructural analysis confirmed the presence of uniformly distributed, interconnected pores throughout the scaffold architecture. Such interconnected porosity is a critical feature for effective cell attachment, proliferation and migration, as well as for enhanced nutrient transport and waste removal. Moreover, the presence of an interconnected pore network is expected to promote vascularisation and bone ingrowth, thereby improving the overall biological performance of the scaffolds.

### 3.2. Investigation of In Vitro Degradation Behaviour: Degradation Rate and pH

**FIGURE 5:**
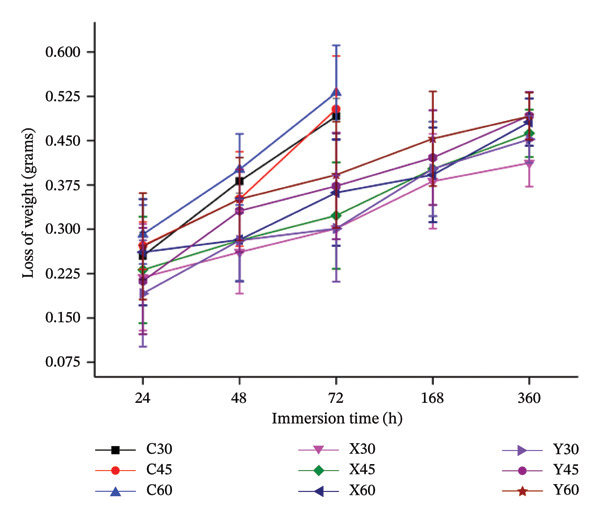
Plot between the decrement in weight of the immersed sample against the sample immersion time.

Figure 5 depicts the variation between sample immersion time and the decrement between the pre‐ and post‐immersion of the biocomposite sample due to its biodegradation in the physiological solution for the required time lines.

**FIGURE 6:**
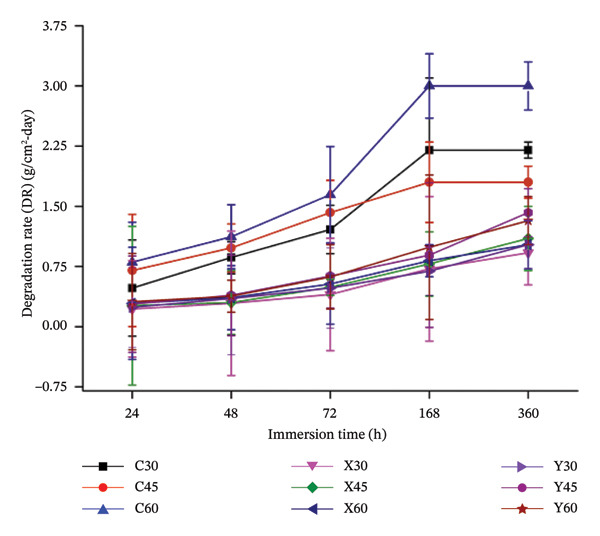
Plot between the rate of degradation of the submerged sample against the sample immersion time.

From Figure 5, it can be noted that the weight change, before and after immersion of the control samples (C30, C45 and C60), is higher as the time line proceeds. Initially, after 24 h of immersion time, the loss of weight of the control samples, of all the three porosities, is lower. This could be due to the fact that the possible formation of the Mg(OH)_2_ is higher, which forms a thin layer over the sample, thereby preventing any further contact with the physiological solution, thus controlling the further degradation, as indicated by equations (2), (3), (4). However, the presence of the chlorine ions in the physiological fluid would react with the newly formed Mg(OH)_2_ and result in highly dissolvable MgCl_2_, thus revealing the Mg and in turn resulting in degradation, which would increase the change of weight [28, 29]. This mechanism explains the higher loss‐of‐weights, especially for the control samples (C30, C45 and C60). Increased porosity would also expose more surface area of the Mg sample to the immersed fluids, thereby promoting the degradation further. Hence, porosity plays a vital role in the degradation. It could be observed that a higher porous sample, that is, 60% porosity sample, has been noted with large variation in the pre‐ and post‐immersion weights, which would be noted from Figure 6. Equations (2)–(4) [30, 31] represent the degradation behaviour of magnesium in aqueous solution:

(2) Mgs+2H2O⟶MgOH2s+H2g

(3) Mg+2Cl−⟶MgCl2

(4) MgOH2s+2Cl−aq⟶MgCl2+H2g

On the other hand, the change of weights, pre‐and post‐immersion for the Mg‐alloyed samples (X and Y) of all three porosities, can be observed from Figure 5. As stated in Table 1, composition X comprises calcium, zinc, strontium and nano‐fluorcanasite as the alloying elements in the Mg metal matrix for all three porosities (30%, 45% and 60%). It can be noted from Figure 5 that the loss‐of‐weight in composition X is lower, than, when compared to composition Y, for all porosities, as the immersion time precedes. This trend in the composition in X could be attributed towards the inclusion of calcium of 1 wt%, in Mg metal matrix, that could have resulted in the limited formation of Mg_2_Ca, thereby encouraging the resistance towards degradation [30, 32, 33]. Zn is attributed to the formation of Mg_2_CaZn_6_, which is a stable intermetallic phase, that retards the degradation and improves corrosion resistance. However, in composition Y, owing to the inclusion of 3 wt % Ca, this could have enhanced the intermetallic phases of Mg_2_Ca, which might have an adverse effect on degradation as more quantities of Mg_2_Ca could promote pitting corrosion [30, 34, 35]. Figure 6 depicts the variation in the rate of the degradation of the fabricated Mg‐alloy‐based bio‐nanocomposite porous scaffold specimens against the sample immersion time.

**FIGURE 7:**
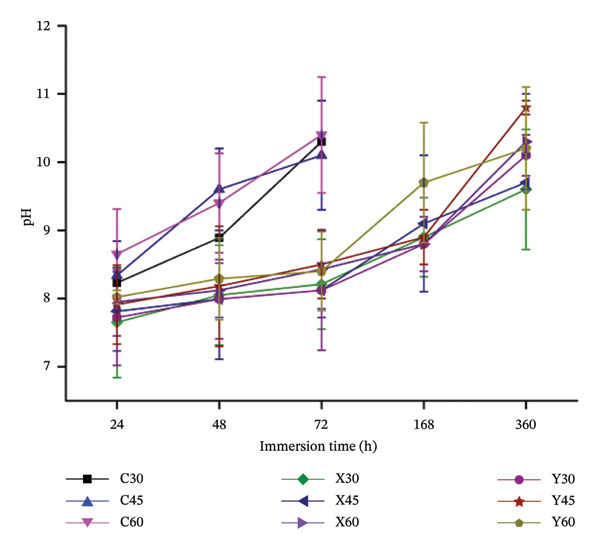
Plot between the pH of the immersed sample against the sample immersion time.

As discussed in the above section, the loss‐of‐weight in the control samples was observed to be highest when compared with the alloyed (X and Y) sample compositions. In the control samples (C) of all the three porosities (30%, 45% and 60%), from Figure 6, the rate of degradation was noted to be lower, post 24 h of sample submersion in PBS solution. Formation of a passive protective layer of magnesium hydroxide on the sample surface prevents any interaction of the physiological solution with Mg, thus limiting the degradation. Free Cl^−^ ions present in the PBS solution would readily react with the magnesium hydroxide and form dissolvable magnesium chloride, which exposes Mg and encourages degradation. But, in the case of the Mg‐alloyed specimens (X and Y), the rate of degradation was controlled post 72 h of degradation, as observed in Figure 6. This trend could be due to incorporation of alloying elements into the Mg metal matrix, which could enhance the resistance towards degradation and also enhance the mechanical attributes of the samples. It was noted that, due to rapid degradation, the control samples (C) were noted to disintegrate post 72 h of sample immersion time. This could be observed in Figure 5, 6 and 7. The change in weights/degradation/pH of the control samples of all the three porosities was noted to be higher, at 72 h of immersion. However, the degradation of the alloyed samples (X and Y) was noted to be controlled post 72 h of sample immersion due to the effect of the alloying elements and also due to the incorporation of the novel bioactive glass–ceramic. Composition X exhibited better degradation behaviour, when compared to composition Y, as the weight percentage of Ca in X was limited to 1 wt%, which could have resulted in the formation of Mg_2_Ca, in limited quantities, as compared to Y composition [18, 36–38]. Although the other alloying elements would also contribute to enhancing the resistance towards degradation, calcium is considered very vital in limiting the corrosion rate in the Mg metal matrix [9, 39]. Figure 7 illustrates the plot between the pH of the sample immersion fluid, against the time elapsed during sample submersion. The results obtained from the pH study were found to be in line with the results obtained during the degradation study. The pH values of the control samples were found to be increasing, as the specimen submersion time elapsed, which is an indication for its rate of degradation. As discussed, due to uncontrollable degradation of Mg in the physiological solution, at 72 h of immersion, the control samples degraded completely, hence, rising the pH to around 11.5. It was also noted that sample porosity plays a vital role in the pH of the immersion liquid, which was a similar trend in the case of degradation study. In the case of the alloyed samples, the pH was noted to be controlled during the initial stages of the immersion. X30 exhibited better degradation behaviour, especially when its rate of degradation and pH of the submerged liquid were lower when compared to the other composition porosities.

Calcium is a natural intergradient of a human bone and also acts as a grain refiner, which is incorporated into the Mg metal matrix. Introducing up to 2 wt % of Ca into the Mg–Zn metal matrix would enhance the formation of Mg_2_Ca, which is a stable intermetallic phase that could be responsible for improving the resistance towards degradation [16, 40, 41]. On the other hand, Zn is a very vital alloying element, as it has the responsibility to control the formation of hydrogen gas, when the Mg is exposed to physiological solution [42, 43]. Large amounts of hydrogen gas liberation near the area of the implanted site could adversely affect the osteoblast and osteocytes, thereby hindering the regrowth of the new bone cells. Zn, which when incorporated into the Mg metal matrix, would be responsible for the formation of Zn(OH)_2_ over the sample surface [44]. This zinc hydroxide acts as a better and strong protective layer, which when compared to the hydroxide formed base of magnesium [44]. Hence, by incorporation of Zn into the Mg–Ca metal matrix would not only enhance the degradation resistance but also improves its mechanical performance, as it contributes in solid solution strengthening [45, 46]. Strontium (Sr) naturally, promotes and encourages for the development of new bone cells, as it is a bone‐seeking element [47]. When Sr is involved into Mg–Ca–Zn in very small amounts, up to 0.5 wt%, it would induce the osteoconductive feature into the Mg–Ca–Zn–Sr intermetallic compound [44]. Introducing a novel bioactive ceramic, which is a nano‐fluorcanasite, would introduce the bioactive nature into the scaffold specimen [48].

### 3.3. Investigation of In Vitro Degradation Behaviour: Hydrogen Gas Emission

**FIGURE 8:**
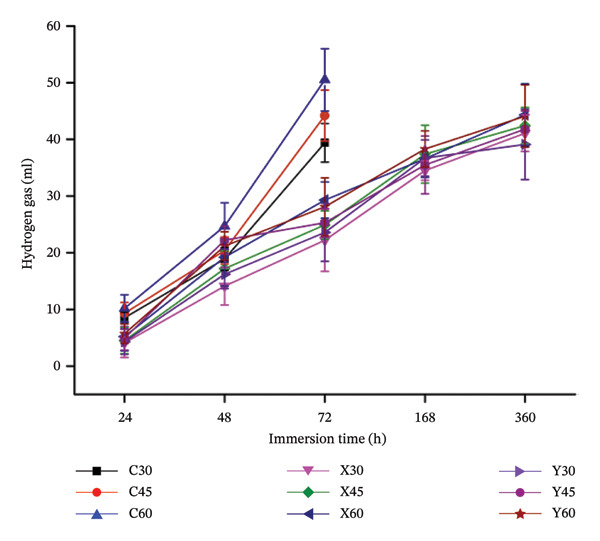
Plot between the hydrogen emission from the immersed scaffold against the sample immersion time.

Figure 8 illustrates the variation between the emission of the hydrogen gas, which is one of the by‐products during the composite sample degradation, against the sample immersion time.

As indicated in equations (2)–(4), the proportional amounts of magnesium, when in contact with aqueous solution, release equal proportional amounts of hydrogen gas. Hence, emission of hydrogen gas, from the immersed magnesium‐alloy‐based bio‐nanocomposite porous samples in PBS solution, is an indication of its corrosion in physiological solution. In vitro and in vivo behaviours of the scaffolds are greatly influenced by the rate of hydrogen evolution, as higher amounts of hydrogen act at the vicinity of the implanted site, which could potentially hamper cell proliferation and cell growth [49, 50]. From Figure 8, it could be noted that for the control samples, (C), all the three porosities (30%, 45% and 60%) exhibit higher amounts of hydrogen gas from the immersed scaffold samples. As discussed in the earlier sections, owing to the existence of the well‐connected pores of different sizes, shapes and orientations in the porous samples, this could lead to higher porosities in the samples that could lead to enhanced rate of degradation, as it provides higher surface area interaction with the physiological solution. Similarly, the same trend was noticed, where higher porosities in the samples lead to enhanced rates of hydrogen gas emission. On the other hand, the hydrogen gas emission from the magnesium‐alloyed samples (X and Y) of all three porosities was observed to be on lower values, post 360 h of immersion. This trend could be owed to the incorporation of the alloying elements, Ca, Zn and Sr. In the composition X that includes 1 wt % of Ca, 2.5 wt % of Zn and 0.5 wt % of Sr, this could have resulted in lower amounts of stable intermetallic compounds such as Mg_2_Ca, MgZn_2_, Mg_2_CaZn_6_ and Mg_2_Sr_17_ that could have improved the degradation behaviour, as the resistance towards degradation could have been enhanced [51, 52]. From Figure 8, it could be observed that the results of the hydrogen gas are in accordance with the results obtained during the degradation study of the same control and Mg‐alloyed samples. Owing to the presence of alloying elements such as Ca, Zn and Sr in the Mg metal matrix could result in the possible development of the hydroxides of the incorporated alloying elements, which could retard the degradation; thereby, the emission of reduced hydrogen gas was compared with that of the control Mg samples [25, 53, 54].

## 4. Conclusions

The powder metallurgy method was effectively used to create magnesium‐alloy‐based bio‐nanocomposite open porous bone implant samples. Calcium, zinc and strontium were included into the magnesium metal matrix at specified weight percentages. A novel bioactive glass–ceramic, fluorcanasite nanoparticles, was integrated into the magnesium metal matrix to enhance and promote the bioactive properties in the magnesium‐alloy specimens.

• A natural human bone comprises of cortical bone and cancellous bone, of which the levels of porosity range up to 60%; hence, the porosity of the produced samples was controlled and examined for a porosity level of 30%, 45% and 60%.
• Alloying elements (Ca, Zn and Sr) that were introduced into the magnesium‐metal matrix could have produced stable intermetallic compounds, such as Mg_2_Ca, MgZn_2_, Mg_2_CaZn_6_ and Mg_2_Sr_17,_ which could have encouraged resistance towards biodegradation.
• pH values of the scaffold immersion fluid and the hydrogen gas evolution from the immersed samples were noted to be in line with the results of degradation behaviour.

Based on the outcomes of this research investigation, the findings indicate that the reengineered magnesium‐alloy‐based composite implant samples may serve as a degradable material for bone tissue repair and reconstruction in orthopaedics and healthcare.

## Author Contributions

Study conception and design: Adithya Garimella, Subrata Bandhu Ghosh and Sanchita Bandyopadhyay‐Ghosh; data collection: Adithya Garimella; analysis and interpretation of results: Adithya Garimella, Subrata Bandhu Ghosh and Sanchita Bandyopadhyay‐Ghosh; draft manuscript preparation; Adithya Garimella, Firoz Alam Faroque, Subrata Bandhu Ghosh and Sanchita Bandyopadhyay‐Ghosh.

## Funding

The work was supported by the ‘Seed Grant’ provided by the Manipal Academy of Higher Education (MAHE), Manipal, India.

## Disclosure

All authors reviewed the results and approved the final version of the manuscript.

## Conflicts of Interest

The authors declare no conflicts of interest.

## Data Availability

All the information/data necessary for the work would be available on request from authors.
